# Formulation and Physicochemical Evaluation of Lab-Based *Aloe adigratana* Reynolds Shampoos

**DOI:** 10.1155/2020/6290617

**Published:** 2020-04-04

**Authors:** Desta Berhe Sbhatu, Goitom Gebreyohannes Berhe, Abadi Gebreyesus Hndeya, Haftom Baraki Abraha, Asmael Abdu, Haftay Abadi Gebru, Molla Gereme Taye, Afework Mulugeta, Micheale Yifter Weldemichael, Hailekiros Tadesse Tekle, Haileselassie Gebremeskel Kidanemariam

**Affiliations:** ^1^Mekelle University, PO Box 231/1632, Mekelle, Ethiopia; ^2^Tigrai Biotechnology Center Pvt. Ltd. Co., Mekelle, Ethiopia; ^3^Mekhoni Agricultural Research Center, PO Box 71, Mekhoni, Ethiopia

## Abstract

*Aloe* L. species (Aloaceae) are ethnobotanically very valuable plants in many communities and civilizations. Nonetheless, very few species are extensively studied to explore their applications in the pharmaceutical and medical, cosmetic and personal care, food and beverage, and detergent industries. This study evaluated the characteristics and quality of lab-based shampoos formulated from the gel of *Aloe adigratana* Reynolds. Five shampoo formulations, 20 mL each, were prepared from *A. adigratana* gel in combination with one to two drops of coconut oil, jojoba oil, olive oil, pure glycerin oil, lemon juice, and vitamin E. Gel mass is prepared from mature, healthy leaves collected from the natural stand. The phytochemistry of the gel of the plant was also studied using phytochemical screening, proximate composition, and GC-MS analysis studies. Shampoo formulations with higher proportion (40 to 50% v/v) of *A. adigratana* gel were found to have comparable characteristics and qualities with a marketed shampoo. They fall within the range of acceptable quality parameters of commercial shampoos. The phytochemical studies of *A. adigratana* gel showed that the plant is the source of highly valued compounds for the preparation of shampoos. The gel was found to be rich in saponins as well as dodecanoic acid, hexadecanoic acid, and phytol. Future works should focus in the development of refined protocol towards formulating *A. adigratana*-based shampoos.

## 1. Introduction

*Aloe* L. (Aloaceae) species are regarded as lilies of the desert, plants of immortality, and medicine plants [1]. They are native to Africa, the Mediterranean region of Southern Europe, South and Central America, Rio Grande Valley of South Texas, Florida, Southern California, Mexico, Pacific Rim countries, India, Caribbean, Arabian Peninsula, and Australia [2, 3]. Ethiopia and Eritrea are home to 50 known and described species [4–7].

Nearly, all species of *Aloe* exhibit high-degree endemism and many of them are restricted to a very small area. Over three-quarter of the Ethiopian aloes are endemic and restricted to few floristic regions and limited habitats [8]. Nine of the 50 Ethiopian and Eritrean aloes grow in the Tigray floristic region, namely, *A. adigratana* Reynolds, *A. camperi* Schweinfurth, *A. elegans* Todaro, *A. macrocarpa* Todaro, *A. monticola* Reynolds, *A. percrassa* Todaro, *A. sinana* Reynolds, *A. steudneri* Schweinfurth, and *A. trichosantha* subsp. *trichosantha*. The most abundantly growing aloes in the Tigray floristic region are *A. adigratana* and *A. elegans* where the former belongs to the Tigray floristic region only. *A. adigratana* grows in rocky places, mostly sandstone or basement complex, between 2,000 and 2,700 masl. Its main flowering period is from January to April. It has one (if erect) to two (if decumbent) meters long stem [5].

Aloes are all-purpose plants recorded in the annals of history of early civilizations. They are used for producing cosmetic and personal care, detergent and toiletry, medicinal and pharmaceutical, and food and beverage products. They are grown for ornamental purposes and large-scale horticulture [9–13]. Herbal formulations of shampoos, personal care, and toiletry products have long been considered as better alternatives to synthetic ones [14–21]. Aloes are important sources of herbal inputs in producing cosmetic, personal care, and toiletry products. However, only *A. barbadensis* Miller (*A. vera* L.) is extensively used in producing lotions, soaps, shampoos, creams, and facial cleansers, see, e.g., [9, 22]. This study aims at describing the physicochemical characteristics of lab-based *A. adigratana* gel shampoo formulations. The plant grows widely in the Tigray floristic region of the Flora of Ethiopia and Eritrea across various ecological conditions. It is planted as fence of farm plots, backyards, churchyards, and footpaths. Unfortunately, the plant is put in the IUCN (International Union for Conservation of Nature) Red List because is it endangered. Thus, studies on the chemistry of this aloe and its shampoo formulations are some of the initiatives aiming at exploring its potential while ensuring its conservation and sustainable use.

## 2. Materials and Methods

### 2.1. Collection and Preparation of Plant Specimens

Healthy and mature leaves of *A. adigratana* were collected from the wild stand in Al'asa (30 km on the Mekelle-Abbiyi Addi highway; lat./long.: 13.681/39.264; alt.: 2,436 m) on 30 March 2019. Collection of biological materials by natives (Ethiopians) for research and development is granted by Article 15, Clause 1 of the Access to Genetic Resources and Community Knowledge and Community Rights Proclamation of Ethiopia (No. 482/2006). Specimens of the plant were identified by the National Herbarium (ETH), the Department of Biology, Addis Ababa University (Ethiopia). The leaves were washed with tap water to remove dirt and soil. The outer green skin (i.e., the leaf) and the inner gelatinous mass (i.e., the gel) were separated by peeling the skin off with scalpel. The gel mass was dried in shade at room temperature for 18 days. The dried gel mass was then pulverized into powder in an electrical grinder and stored in sealed container until used for the phytochemical study.

### 2.2. Gross Phytochemistry of Gel Extracts

The *A. adigratana* gel powder was extracted by 100% methanol using the continuous hot percolation method in a Soxhlet apparatus for 18 hours. The extract was concentrated in a rotary evaporator to yield a brown liquid. The extract was kept at 4°C in a deep freezer. Then, samples of the extracts were subjected to preliminary phytochemical screening using standard tests for alkaloids (Wagner test), anthraquinones (Borntrager's test), flavonoids (lead acetate test), saponins (froth test), tannins (ferric chloride test), and terpenoids (Salkowski test) [23–27]. Besides, some gel extract was subjected to esterification and gas chromatography mass spectroscopy (GC-MS) at JIJE LOBOGLASS Pvt. Ltd. Co., Addis Ababa, Ethiopia. Instrument control parameters of the GC-MS are given in Annex 1.0.

### 2.3. Formulation of *A. adigratana* Gel Shampoos

*A. adigratana* shampoos were prepared from gel mass by mixing it with six ingredients, namely, coconut oil, jojoba oil, lemon juice, olive oil, pure glycerin oil, and vitamin E (Table 1). Five shampoo formulations were prepared by mixing the ingredients at varying concentrations (amounts) (Table 2) and homogenizing the contents by a mechanical stirrer [28, 29]. The volumes of all the formulations were fixed at 20 mL by adding sterile distilled water.

### 2.4. Evaluation of the Characteristics of the Shampoos

The five shampoo formulations were physically evaluated by inspecting and measuring their color, clarity, odor, consistency, spreadability, and pH at 25°C. Likewise, the qualities of the formulations were evaluated by analyzing their solid contents, surface tension, dirt dispersion, rheology (viscosity) (Model DV-l Plus, LV, USA), foaming stability, wetting time, and conditioning performance based on the procedures established by many researchers, e.g., [15, 23, 24, 28, 29].

## 3. Results and Discussion

### 3.1. Gross Phytochemistry of *A. adigratana* Gel

*A. adigratana*, like all aloes, can be the source of many phytochemical constituents applicable in preparing cosmetic, pharmaceutical, and many other products. Preliminary phytochemical screening of methanol gel extracts using standard tests showed the presence of anthraquinones, flavonoids, saponins, and tannins and the absence of alkaloids and terpenoids (Table 3). Brhane et al. [30] used multiple extraction solvents and reported the presence of alkaloids, flavonoids, tannins, saponins, polyphenols, and terpenoids with *in vitro* antioxidant properties. Leaf latex of the plant is also reported to be the source of two anthrones with anti-inflammatory activities [31]. Besides, proximate analysis exhibited that the moisture and ash content, crude fat, total protein, and carbohydrate of the plant's gel were 92.19 ± 0.03%, 3.51 ± 0.01%, 0.24 ± 0.04%, 1.64 ± 0.09%, and 2.61 ± 0.07%, respectively [32]. In fact, there are many plants species that produce useful chemical constituents for hair care such as vitamins, amino acids, sugars, glycosides, phytohormones, bioflavonoids, fruit acids, and essential oils, thus commonly used in the formulation of shampoos [16].

GC-MS analysis of the gel extract revealed that it has 13 compounds (Table 4). Many of the compounds are used in making beauty and personal care products. Decanoic (capric) acid (**2**), dodecanoic (lauric) acid (**3**), hexadecanoic (palmitic) acid (**8**), n-hexadecanoic acid (**9**), (Z, Z)-9, 12-octadecadienoic (linoleic) acid (**10**), and phytol (**13**) are used in formulating personal care products including soaps and detergents. Decanoic (capric) acid (**2**), tetradecanoic (myristic) acid (**7**), hexadecanoic (palmitic) acid (**8**), (Z, Z)-9, 12-octadecadienoic (linoleic) acid (**10**), and phytol (**13**) are employed in producing cosmetics and beauty products. Octadecanoic (stearic) acid (**12**) and phytol (**13**) are also used in producing shampoos and shaving creams. Furthermore, whereas compound **10** is an important source of surfactants, compound **12** is used in saponification [33].

### 3.2. Evaluation of *A. adigratana* Gel Shampoos

#### 3.2.1. Sensory Assessment

Good shampoos have attractive appearance to the sensory observer like the case with all cosmetic products. As indicated in Section 2, *A. adigratana* shampoos were formulated by mixing gel masses of the plant with some amounts of synthetic and natural ingredients (Tables 1 and 2). The formulations were maintained at pH of 6 ± 0.4 in compliance to skin health and safety regulations. Then, they were evaluated by comparing each of them against one commercial shampoo through sensory observation and simple measurements, namely, color, clarity, odor, consistency, spreadability, pH, and temperature. The formulations were transparent and light-green with good odor. They demonstrated no significant difference from the commercial shampoo in terms of odor, transparency, and foaming characteristics except color (Table 5). Since no coloring agent was added, the formulations were white. Varying the proportion of *A. adigratana* gel did not lead to change in color, turbidity, and characteristic odor.

Consistencies of the formulations changed from thin to slightly thick with doubling the proportion of the gel from 4–6 mL to 8–10 mL. The spreadability was found to be the best at 6 to 10 mL of gel. Increasing the proportion of the gel led to a slight increase in pH from 6.4 (for 2 and 3 mL) to 6.8 (for 5 mL) at 25°C. Their pH values fall within the pH range of many commercial shampoos that often set from 6.0 to 7.0 at 25°C [28, 29, 34]. Most commercial shampoos are formulated as either neutral or slightly alkaline to minimize hair damage, enhance hair quality, minimize eye irritation, and maintain the ecological balance of the scalp [15, 28, 29, 35]. With the exception of color, formulations F_3_ to F_5_ have very similar physical features to the commercial shampoo used for this study and other commercial shampoos tested elsewhere [28].

#### 3.2.2. Quality Characteristics

*(1) Solid Content*. The qualities of the formulated shampoos were evaluated using some physicochemical parameters, namely, solid content, foam stability, dirt dispersion, surface tension, wetting time, and conditioning performance (Table 6). Solid content is one of the physical parameters used in establishing the quality of shampoos. Lower solid content makes shampoos watery and drain off hair quickly, while higher solid content makes them difficult to work with and rinse off the hair. The solid contents of our formulations ranged from 23 to 28%. They were easy to apply to the hair and rinse off. Solid content of 20 to 30% is considered as the preferred attribute of commercial shampoos. Many researchers were able to formulate herbal shampoos with solid content falling within the acceptable range [28, 36–38].

*(2) Foam Ability and Stability*. Volume and stability of foams are also principal parameters in assessing the quality and consumer acceptance of shampoos [39]. Shampoos with big (as expressed in terms of volume) and stable foams (as expressed in the length of time they maintain their volumes) are regarded as the most preferred. Good shampoos produce big volume of stable foams after shaking. *A. adigratana* shampoos with 8 to 10 mL of gel produced foam volumes comparable to that of commercial *A. vera* shampoo used in the study. The foams of all formulations were compact, uniform, and stable like that of the commercial one. They maintained the same volume for five minutes. One study by Al Badi and Khan [28] with commercial dove shampoo and formulated herbal shampoo reported similar results.

*(3) Dirt Dispersion*. Dirt dispersion is another key parameter in evaluating the cleansing action of shampoos, whereas high-quality shampoos concentrate the dirt in the water, poor-quality ones concentrate the dirt in their foams. Any dirt or stain that concentrates in the foam is difficult to rinse away and can be redeposited on the hair. Shampoos that concentrate the dirt or stain in the water have good cleaning ability [28, 29, 40]. All our formulations yielded clean foams and water concentrated with dirt. No dirt was observed in the foams of all formulations. Similar findings were reported with herbal shampoos of plants [36].

*(4) Surface Tension*. The present study resulted in shampoo formulations with measures of surface tension ranging from 33 (for formulation with 10 mL gel) to 38 dynes/cm (for formulation with 4 mL gel). Preferred shampoos are those that reduce the surface tension of pure water from 72 dynes/cm to less than 40 dynes/cm at 25°C [41]. Many other researchers were able to formulate herbal shampoos with surface tension between 30 and 40 dynes/cm, e.g., [36, 38]. The effectiveness of shampoos is affected by the amount of surfactants or any other agents that reduce surface tension of water. Therefore, shampoos that reduce the surface tension of water have good detergency [42]. Apparently, the surface tensions of *A. adigratana* shampoo formulations fall below 40 dynes/cm at 25°C. With 8 to 10 mL of gel, our formulations were found to have comparable surface tension with the commercial shampoo used in this study (Table 6) and other shampoos studied elsewhere [28].

*(5) Wetting Ability*. Wetting abilities of shampoos depend on the concentration of their surfactants. Higher concentrations of surfactants lead to better wetting ability. Canvas disc method is a quick, efficient, and reliable test in evaluating the wetting abilities of shampoos as a function of wetting time [28, 29]. The most preferred shampoos are those that have shorter wetting time. The wetting time of *A. adigratana* shampoo formulations was compared against that of commercial *A. vera* shampoo. Our formulations yielded lower wetting time (142 to 157 sec.) compared to that of the commercial one (185 sec.). Shampoo formulations with shorter wetting time have higher concentrations of detergents [15, 28, 29].

*(6) Conditioning Performance*. The conditioning performances of shampoos depend on their chemistries. Shampoos are enriched with conditioning polymers that deposit, adhere, or adsorb onto the proteins of hairs. The polymers improve hair manageability, reduce static, and make hairs soft and smooth [15, 28, 29]. The conditioning effect of the shampoos was studied by washing a mass of cut hair with the formulations and making physical observations. All our shampoo formulations demonstrated good conditioning performance. The mass of hair was glowing, soft, silky, and manageable.

*(7) Viscosity*. Viscosity of a shampoo is the reflection of the amount of its solid content. Viscosity plays a key role in defining many attributes of shampoos such as their spreadability upon application and consistency in their package [37]. The viscosities of our shampoo formulations were determined using Brookfield Viscometer Spindle No. 2. The viscosity values of the shampoos ranged from 22.19 (4 mL gel) to 26.86 poise (10 mL gel) (Table 7). Shampoo formulations with higher proportions of *A. adigratana* gel showed higher viscosities. Higher viscosity makes shampoos thicker with improved consistency. These characteristics are attributed to the higher proportion of the gel [28]. Our formulations had 95.73 to 95.86% of moisture. They fall within an acceptable range [28, 29].

## 4. Concluding Remarks

Ethiopian aloes have been vital components of ethnomedicine. Studies on phytochemical constituents and medicinal properties of the aloes showed that they are good sources of bioactive compounds [43–48]. Few studies explored the phytochemical properties of *A. adigratana* [30–32]. Our phytochemical screening using the Froth test was positive for saponins—the principal class of phytochemicals with natural surfactants. Moreover, GC-MS analysis of the methanol gel extracts showed that the plant is the source of high amount of many phytochemicals used in the production of cosmetics and personal care products (e.g., dodecanoic acid, hexadecanoic acid, and phytol). The lab-based shampoo formulations of gel extracts prepared and evaluated in the present study exhibited desirable features like the marketed shampoo used for comparison. Sensory observation and physiochemical tests also revealed that the formulations have required qualities. Further studies are recommended to elucidate the complete phytochemical profile of the plant and develop a more refined protocol of shampoo making.

## 5. Annex **1.0**

### 5.1. Instrument Control Parameters of GC-MS

D:\MassHunter\GCMS\1\methods\Fatty Acid_A. Adigratana_DB5MS 10.M Wed Jul 17 11 : 39 : 33 2019; control information: sample inlet, GC; injection source, GC ALS; injection location, front; and mass spectrometer, enabled. GC : oven temperature; set point on, (initial) 60°C; hold time, 0 min; and postrun, 50°C. Program: #1 rate 3°C/min; #1 value 110°C; #1 hold time 0 min; #2 rate 10°C/min; #2 value 140°C; #2 hold time 1 min; #3 rate 5°C/min; #3 value 195°C; #3 hold time 10 min; #4 rate 5°C/min; #4 value 225°C; #4 hold time 6 min; #5 rate 20°C/min; #5 value 250°C; and #5 hold time 4 min.

## Figures and Tables

**Table 1:** Ingredients of component used in formulating *A. adigratana* gel shampoos.

Generic name	Biological applications	Manufacturer
*A. adigratana* gel	Repairs, strengthens, hydrates, softens hair; makes hair look and feel healthier; heals wound; acts as cosmetics	Lab-based formulation
Coconut oil	Prevents protein loss in hair; moisturizes skin; acts as a natural sunscreen	C.B.C., Malaysia
Jojoba oil	Moisturizes and gives hair shining look	ORS, USA
Lemon juice	Acts as natural antioxidant, chelating, and antidandruff agent; maintains the pH of the acidic formulation	Lab-based extract
Olive oil	Moisturizes hair; reduces scalp irritation and dandruff	Salamati, Spain
Pure glycerin oil	Hydrates skin; boosts cell maturation; removes dandruff	LFRESSH-eurogulf, UAE
Vitamin E	Supports scalp; gives hair strong base to grow; reduces oxidative stress; preserves protective lipid layer	Fruit of the earth, USA

**Table 2:** Formulation of *A. adigratana* shampoos.

Ingredients	UoM^*∗*^	Formulations				
		F_1_	F_2_	F_3_	F_4_	F_5_
*A. adigratana* gel	mL	4	6	8	10	10
Coconut oil	Drops	1	1	1	1	2
Olive oil	Drops	1	2	1	2	1
Jojoba oil	Drops	1	1	1	1	1
Glycerin oil	Drops	1	1	1	1	1
Vitamin E	Drops	1	1	1	1	1
Lemon juice	Drops	1	1	1	1	1

Proportion of gel	(%), v/v	20	30	40	50	50

^*∗*^UoM (unit of measurement).

**Table 3:** Proximate composition of *A. adigratana* leaf gel.

Composition	Tests	Inspection	Results^*∗*^	Reference
Alkaloids	Wagner test	Brownish-red precipitate	–	[26]
Anthraquinones	Borntrager's test	Pink, red	+	[24]
Flavonoids	Lead acetate test	Yellow precipitate	+	[25]
Saponins	Froth test	Foam	+	[23]
Tannins	Ferric chloride test	Dark-green	+	[27]
Terpenoids	Salkowski test	Reddish-brown	–	[23]

^*∗*^“ + ” sign indicates the presence and “ – ” sign indicates absence of the chemical constituents.

**Table 4:** Chemical composition of *A. adigratana* leaf gel extract.

SN	Name	Formula (DB)	Area	*t*_R_	% area
**1**	Naphthalene, 1-methyl-	C_11_H_10_	1,232,037	20.70	2.41
**2**	Decanoic acid, methyl ester	C_11_H_22_O_2_	305,685	21.37	0.60
**3**	Dodecanoic acid, methyl ester	C_13_H_26_O_2_	1,655,246	26.54	3.24
**4**	aR-Turmerone	C_15_H_20_O	892,564	29.90	1.75
**5**	Turmerone	C_15_H_22_O	553,201	30.03	1.08
**6**	Curlone	C_15_H_22_O	803,203	30.78	1.57
**7**	Tetradecanoic acid, methyl ester	C_15_H_30_O_2_	4,382,715	31.16	8.59
**8**	Hexadecanoic acid, methyl ester	C_17_H_34_O_2_	10,391,272	36.54	20.36
**9**	n-Hexadecanoic acid	C_16_H_32_O_2_	6.074,844	37.94	11.90
**10**	(*Z, Z*)-9, 12-Octadecadienoic acid, methyl ester	C_19_H_34_O_2_	1,310,901	43.75	2.57
**11**	(E)-9-Octadecadienoic acid, methyl ester,	C_19_H_36_O_2_	7,144,589	44.08	14.00
**12**	Octadecanoic acid, methyl ester	C_19_H_38_O_2_	2,831,471	45.12	5.55
**13**	Phytol	C_20_H_40_O	13,466,480	44.49	26.38

*t*_R_ : retention time.

**Table 5:** Physical inspection of *A. adigratana* leaf gel shampoos.

Formulations	Color	Clarity	Odor	Consistency	Spreadability	*p*H	Temp. (°C)
F_1_ (4 mL)	White	Turbid	Characteristic	Thin	Good	6.4	25
F_2_ (6 mL)	White	Turbid	Characteristic	Thin	Best	6.4	25
F_3_ (8 mL)	White	Turbid	Characteristic	Slightly thick	Best	6.5	25
F_4_ (10 mL)	White	Turbid	Characteristic	Slightly thick	Best	6.6	25
F_5_ (10 mL)	White	Turbid	Characteristic	Slightly thick	Best	6.8	25
Commercial	Green	Turbid	Characteristic	Slightly thick	Best	6.7	25

**Table 6:** Evaluations of *A. adigratana* shampoo formulations.

Formulation	Solid content (%)	Foam stability	Dirt dispersion	Surface tension	Wetting time test	Conditioning performance	Temp. (°C)
F_1_ (4 mL)	23	Good	Not detected	38	142	Good	25
F_2_ (6 mL)	24	Good	Not detected	37	150	Good	25
F_3_ (8 mL)	26	Very good	Not detected	36	152	Good	25
F_4_ (10 mL)	28	Very good	Not detected	34	153	Good	25
F_5_ (10 mL)	25	Very good	Not detected	33	157	Good	25
Commercial	26	Very good	Not detected	32	185	Good	25

**Table 7:** Viscosities of *A. adigratana* gel shampoos.

Formulations	Viscosity (poise)	Speed (rpm)	%FSR	Shear stress	Stress rate	Temp (°C)
F_1_ (4 mL)	22.19	60	69.17	18,672.23	899.99	25
F_2_ (6 mL)	24.09	60	68.70	186,112.11	899.99	25
F_3_ (8 mL)	24.11	60	67.19	165,112.17	899.99	25
F_4_ (10 mL)	26.17	60	66.17	168.88	899.99	25
F_5_ (10 mL)	26.86	60	69.12	169.82	899.99	25

## Data Availability

The datasets used and/or analyzed during the current study are available from the corresponding author on reasonable request.
